# Neurobiology and Anatomy of Facial Expressions in Great Apes: Application of the AnimalFACS and Its Possible Association with the Animal’s Affective State

**DOI:** 10.3390/ani14233414

**Published:** 2024-11-26

**Authors:** Adriana Domínguez-Oliva, Cuauhtémoc Chávez, Julio Martínez-Burnes, Adriana Olmos-Hernández, Ismael Hernández-Avalos, Daniel Mota-Rojas

**Affiliations:** 1 PhD Program in Biological and Health Sciences, Universidad Autónoma Metropolitana (UAM), Mexico City 04960, Mexico; 2 Neurophysiology of Pain, Behavior and Animal Welfare Assessment, DPAA, Universidad Autónoma Metropolitana (UAM), Mexico City 04960, Mexico; 3 Departamento de Ciencias Ambientales, CBS, Universidad Autónoma Metropolitana-Lerma, Lerma de Villada 52005, Mexico; 4 Facultad de Medicina Veterinaria y Zootecnia, Universidad Autónoma de Tamaulipas, Victoria City 87000, Mexico; 5 Bioterio and Experimental Surgery, Instituto Nacional de Rehabilitación-Luis Guillermo Ibarra Ibarra (INR-LGII), Mexico City 14389, Mexico; 6 Biological Sciences Department, Facultad de Estudios Superiores Cuautitlán, Universidad Nacional Autónoma de México, Cuautitlán 54714, Mexico

**Keywords:** western lowland gorilla, Bornean orangutan, chimpanzee, facial action unit

## Abstract

The Facial Action Coding System (FACS) is an anatomically based system to study facial expression in humans. Currently, it is recognized that nonhuman animals, particularly nonhuman primates, have an extensive facial ethogram that changes according to the context and affective state. The facial expression of great apes, the closest species to humans, has been studied using the ChimpFACS and OrangFACS as reliable tools to code facial expressions. However, although the FACS does not infer animal emotions, making additional evaluations and associating the facial changes with other parameters could contribute to understanding the facial expressions of nonhuman primates during positive or negative emotions. The present review aims to discuss the neural correlates and anatomical components of emotional facial expression in great apes. It will focus on the use of Facial Action Coding Systems (FACSs) and the movements of the facial muscles (AUs) of chimpanzees, orangutans, and gorillas and their possible association with the affective state of great apes.

## Simple Summary

In humans, it is suggested that facial expressions reflect the emotional state of the individual but can also serve as a communicative signal. Chimpanzees, orangutans, and gorillas are the species closest to humans. Therefore, studying if nonhuman primate facial expression changes according to the affective state is relevant for research focused on the emotional responses of animals. The present review aims to discuss the neural correlates and anatomical components of emotional facial expression in great apes. It will focus on the use of Facial Action Coding Systems (FACSs) and the movements of the facial muscles (AUs) of chimpanzees, orangutans, and gorillas and their possible association with the affective state of great apes.

## 1. Introduction

Great apes are highly social species that communicate through acoustic, tactile, olfactory, and visual cues [1,2]. For primates, facial displays provide information about the animal’s motivation, intentions, and affiliative state [3,4,5]. Facial expressions are being widely studied in animals as a way to assess the response to negative/positive contexts or emotional valence through facial muscle movements [6,7,8,9]. Nonetheless, although facial expressions have a communicative role, this is highly influenced by the species and their social context [10].

An example of the challenges in associating facial expressions with an emotional context is the so-called facial feedback hypothesis (FFH) [11]. This hypothesis mentions that inhibiting certain facial expressions can attenuate the subjective emotional experience related to facial changes [11]. Humans smile when experiencing pleasant stimuli and frown when feeling sad [12,13]. In Mori et al.’s [14] study, raising the cheeks with bandages elicited a feeling of happiness in the participants, while other authors reported that facial feedback influences only emotions such as happiness, anger, and disgust [12,13,15]. In nonhuman primates, this has not been extensively researched. However, expressing and interpreting facial displays help them to appropriately respond to conspecifics and, possibly, their association with emotional states [1,16].

In humans, to objectively study facial displays, Paul Ekman and collaborators developed and standardized a coding system describing facial movements (AUs) or distinctive movements of facial muscles, according to the underlying action of facial or mimetic muscles, and named it the Facial Action Coding System (FACS) [17,18,19,20]. FACS is an anatomically based system that associates each FAU (e.g., upper lip raiser) to their specific muscle (e.g., *levator labii superioris*) [20,21]. Using the human FACS as a reference, AnimalFACS have been developed in dogs (DogFACS) [22], cats (CatFACS) [23], horses (EquiFACS) [24], rhesus monkeys (*M. mulatta*) (MaqFACS) [25], gibbons (*Symphalangus syndactylus*, *Hylobates pileatus*, *Hylobates moloch*, *Nomascus siki*, *N. gabriellae*, *N leucogenys*, *H. muelleri*) (GibbonFACS) [26], common marmosets (*Callithrix jacchus*) (CalliFACS), and great apes including chimpanzees (*P. troglodytes*) (ChimpFACS) [20] and orangutans (*Pongo pygmaeus*, *Pongo abelii*) (OrangFACS) [27]. The first AnimalFACS was ChimpFACS due to the resemblance of chimpanzees to human faces [20].

The study of facial expressions in great apes has been performed through facial ethograms. The current approaches try to combine AnimalFACS with facial displays frequently observed in primates such as open-mouth faces, silent bared-teeth displays, and alarm faces, among others, to provide an objective interpretation of facial movements and a possible perspective on the facial expression of emotion [28,29]. However, since the emotional face perception in great apes is challenging and not all species have a FACS (e.g., lowland gorilla), the present review aims to discuss the neural correlates and anatomical components of emotional facial expressions in great apes. It will focus on the use of AnimalFACS, specifically the AUs in chimpanzees, orangutans, and gorillas, and its possible association with the affective state of great apes.

## 2. Neurophysiology of Facial Expression in Animals

Facial expressions are being widely studied in animals as a way to assess the response to negative/positive context through facial muscle movements [6,7]. Currently, the term “facial behavior” refers to observable facial movements associated with a species-specific behavioral repertoire [30]. The neurophysiology of facial expression is not completely understood in mammals (including human and nonhuman primates). However, it is known that facial expressions do not depend solely on anatomical components but are the result of a circuit that integrates subcortical and cortical areas such as the amygdala, the primary motor cortex, the ventrolateral cortex, the motor area, the supplementary motor area, two dorsal motor areas of the middle cingulate, and the motor fibers responsible for innervating the facial muscles [10,16].

**Figure 1:**
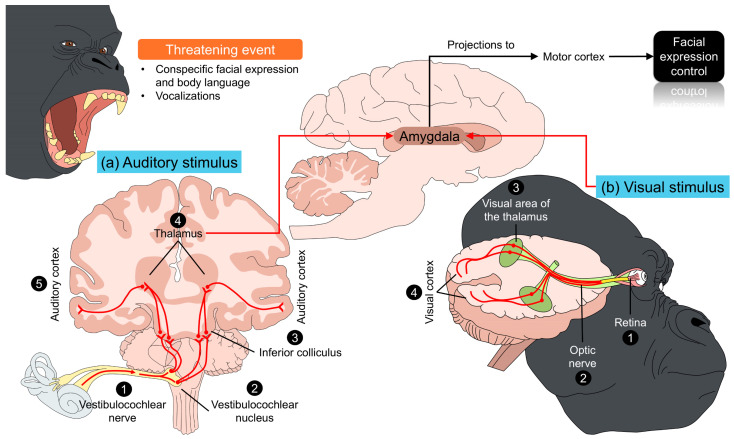
Modulation of visual and auditory stimuli and its influence on facial expression. After perceiving a threatening event (e.g., agonistic interactions with conspecifics), great apes respond to both visual and auditory stimuli. After the integration of both inputs, the connections from the thalamus to the amygdala are the first step in modifying the facial expression. From the amygdala, direct projections to the motor cortex and, subsequently, to the facial nerve modulate facial expression and mimetic muscles.

A proposed order to produce a certain facial expression starts with (1) somatosensory or proprioceptive neurons that send signals of the individual’s state; (2) a consequent connection from these neurons to emotion-related or limbic-related structures; (3) motor cortical neurons that project to last-order motor neurons; and (4) the efferent pathways from last-order motor neurons to mimetic facial muscles [31]. The afferent pathway related to a stimulus depends on its nature. An example is the exposition of an aversive auditory stimulus (e.g., predator vocalization) [32]. Auditory cues are projected from the cochlea to the thalamus, to subsequently project to the amygdala [33,34]. On the other hand, visual stimuli are processed by the thalamus, specifically by the dorsal lateral geniculate nucleus that receives information from the periphery to the amygdala [35]. In monkeys, 12% of the so-called “eye-fixation cells”, a type of specialized neuron in the amygdala, are activated within 80–140 ms during fixated gaze with unfamiliar conspecifics [36]. These processes are schematized in Figure 1 [32,33,34,35].

The perception and production of facial expressions are triggered by neuronal connections in the primate amygdala and midcingulate cortex, discriminating each facial expression into corresponding contexts [31,34]. The hypothalamus also coordinates some of the facial expressions that are present during sexual interactions, as well as other behavioral and physiological changes that can accompany facial expressions (e.g., tachycardia, hyperthermia, and cortisol increases, among others) [37]. In rhesus macaques (*Macaca mulatta*), processing facial expressions from conspecifics has been shown to activate areas in the amygdala and the superior temporal sulcus [38]. Although the amygdala is the main center where emotions are processed, the temporal and prefrontal cortex also process socio-emotional information in the mammal brain [31,39].

Facial expression is coordinated by the cerebral cortex and subcortical motor pathways [31]. Facial movements require motor control of the upper and lower muscles. This modulation comes from the interaction between the ventrolateral frontal cortex, the supplementary motor area, and two areas in the middle cingulate cortex. Last-order motor neurons are responsible for directly innervating facial muscle fibers according to signals from the cortical motor neuron circuit [40]. The amygdala and the middle cingulate cortex are structures that activate during the perception and production of facial expressions, showing that expressions depend on motor and limbic areas of the brain [16].

**Figure 2:**
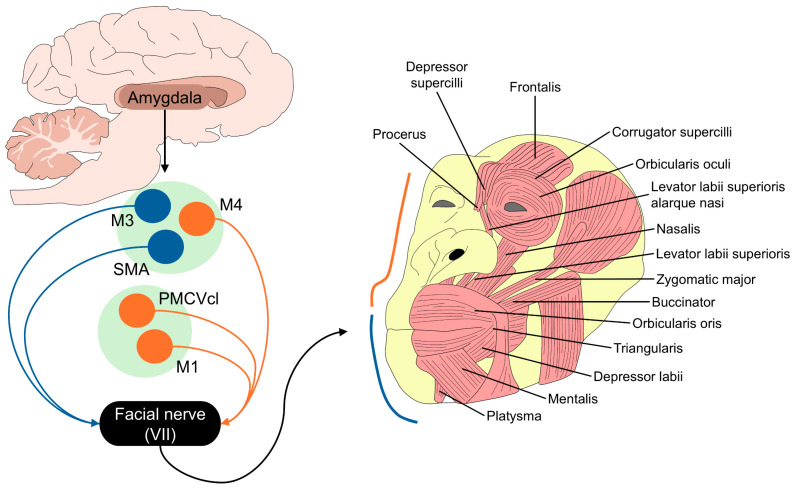
Motor control of facial expression and mimetic muscles in chimpanzees. M1: primary motor cortex; M3: anterior face area of the midcingulate motor cortex; M4: caudal area of the anterior middle cingulate; PMCVcl: premotor cortex ventrolateral division; SMA: supplementary motor area.

At the cortical level, five regions project to the facial nucleus: (1) the primary motor cortex (M1), from which connections arise to the entire facial nucleus, especially to the contralateral lower muscles; (2) the ventrolateral area of the premotor cortex (LPMCv) and the dorsolateral premotor cortex, which innervate the lower facial muscles; (3) the caudal area of the anterior middle cingulate (M4), which innervates the lower facial muscles; (4) the supplementary motor cortex (M2); and (5) the middle cingulate motor cortex (M3) [5,41]. M2 and M3 innervate the upper muscles of the face and are associated with afferents from the limbic system for motor control. These projections go from the amygdala to M3 and from M3 to the facial nucleus, where M3 is known as the major mediator center for the facial expression of emotions such as fear or happiness [41]. This neurobiological control is schematized in Figure 2, along with the facial muscles in chimpanzees [31,42].

The motor nucleus of the facial nerve is the largest in the brain stem. In humans, there are 24 facial muscles [43], but 17 are considered mimetic muscles [41]. In the case of nonhuman primates, 16 mimetic muscles have been reported in chimpanzees (*Pan troglodytes*) [21,42] and gorillas [4]. These mimetic muscles share innervation by the facial nerve, as well as a similar embryological origin and the absence of muscle spindles, which means that they lack stretch reflexes, requiring the process and integration of cutaneous receptors by the central nervous system to produce facial movements [41]. The extracranial facial nerve (along with its distal branches and the trigeminal nerve), a purely motor nerve, stimulates muscle movements. In contrast, sensory information is captured by mechanoreceptors in the facial skin [44].

Motor control of the lower face is coordinated by three motor areas: M1 or the primary motor cortex, the ventrolateral premotor cortex, and the caudal aspect of the middle cingulate cortex. On the other hand, the upper part depends on two motor areas: the supplementary motor area and the anterior aspect of the middle cingulate cortex [45]. Facial paralysis models have shown that contralateral paralysis is observed in the lower part of the face, meaning that the lower part of the face is innervated unilaterally and contralaterally, while the upper part has bilateral innervation [41].

To objectively identify specific muscle movements associated with certain events (e.g., pain, sadness, anger, fear, and disgust) [46], Ekman and Friesen [47] developed the Facial Action Coding System (FACS) in humans. The FACS describes 44 AUs in humans, which represent the activation of a muscle, for example, the *medial* or *lateral frontalis* to raise the eyebrow or the *orbicularis oculi pars palpebralis* to squint (AU44), and the depression of the eyebrows (AU4) is the combined action of the *corrugator supercilli* and *procerus* muscles or closing the eyes (AU43) [17,21,45]. The anatomical understanding of the FACS allows its application to different fields, including veterinary medicine, where the study of facial expression is gaining importance as a method to recognize animals’ behavioral and emotional responses.

## 3. Facial Expression in Nonhuman Mammals and AnimalFACS

Darwin was the first to mention that nonhuman animals can show emotions through facial expression, which constitutes an innate, adaptive, and evolutionarily conserved response [48]. In the last decade, the study of facial expression began to have more importance in the case of animals, particularly as a method to recognize the mental state of animals when exposed to certain stimuli [49].

As Darwin describes, facial expression can be assessed under three categories: (1) descriptive; (2) functional; and (3) causal [50]. Description often refers to the facial musculature of animals and can be composed of AUs, as discussed below [30,51]. Function refers to the intention of the facial expression. For example, in nonhuman primates, it could be to promote cohesive, friendly, playful, or aggressive responses to conspecifics [52,53].

This has been observed in macaques and great apes with the relaxed open-mouth (ROM) expression, often called “play face” (PF) because it is known as a reciprocal interaction during maternal interactions or play fighting [54]. This has been studied by observing interactions between Japanese macaques, in whom PF initiated play bouts and increased the duration of the playtime [55]. Similarly, in another primate species (*Theropithecus gelada*), PF and full PF (an expression where the individual exposes both the lower and upper teeth) were evaluated in juvenile and adult monkeys. In this study, it was reported that PFs are mainly performed by juvenile subjects but that adult animals were more sensitive to respond to PF, probably due to their previous playful experience and social maturity [56].

On the other hand, causes or determinants of facial expression comprise an array of interacting factors that determine the change [50]. Following the example of the PF, primates display ROM during dyadic encounters to convey to the playmate that the interaction is playing and that it has no intention of escalating to aggression [57,58,59].

The study of animal facial expressions has been used as a method to recognize the mental state of animals when exposed to certain stimuli, particularly to negative experiences such as pain [8]. This led to the development of “grimace scales” or scales focusing on the facial expressions that animals show when experiencing pain [6]. Validated grimace scales have been developed in several species such as rodents [60,61,62], sheep [63], horses [64], ferrets [65], piglets [7,66], pigs [67], rabbits [68], and cats [69,70]. Moreover, initial evaluations in marine mammals (*Phoca vitulina*) [71] and dogs [9,72] aim to evaluate the degree of pain in these species. For example, the grimace scale developed for rats has characterized the animal’s pain-face as a narrowing of the orbital area, flattening of the cheeks, ears angled forward, and stiff whiskers by performing observations during the postsurgical period or after the administration of inhalant anesthetics [62,73]. Likewise, in horses experiencing dental disorders without analgesic treatment, observations of the animal’s grimace determined that a facial expression of severe pain is denoted by lowered ears, contraction of the muscle above the eyes, dilated nostrils, tension of the facial muscles, and a muzzle with an edged shape [74].

Although some studies aim to identify changes in facial expression to assess the welfare and mental state of animals [75,76], studying facial expressions in animals is challenging because the same facial expression might differ across species and have different descriptions, functions, and causes [50]. To address this issue, a comprehensive and anatomically based system to distinguish all possible visible facial movements [17,47] has been adapted to animals: AnimalFACS.

FACS assists in the investigation of behavior and gestures, as well as their association with social context and animal interactions with conspecifics or objects [75]. It is noteworthy that the AnimalFACS is not an ethogram of facial expressions, and by solely using these systems, it is not possible to understand an animal’s emotional state. However, using the FACS to assign certain AUs to a facial expression and translate these to a practical environment could help to objectively associate the facial expression with a positive/negative environment. Furthermore, although the FACS is an objective assessment method to anatomically describe facial movements, a disadvantage is that it is based on clearly visible AUs, so subtle changes are not considered, and other facial phenomena such as skin coloration, tears, or sweating are also excluded [77].

## 4. AnimalFACS Systems Focused on Great Apes

Primates rely on facial expressions to communicate with conspecifics in their social networks [78]. Due to this, primates have quite complex facial musculature, involving perioral and periocular movements, producing a wide range of facial expressions [28,43]. Some authors mention that great apes use lips and mouth movements more frequently than orbital or auricular ones leading to the interest in studying their facial expression and their facial anatomy [79].

Currently, two AnimalFACS focusing on great apes have been developed. Both ChimpFACS and OrangFACS use facial muscle contractions (or AUs) to describe facial movements. Most AUs refer to the contraction of single mimetic muscles—muscles innervated by the facial nerve [80]—but some muscles are capable of producing different movements and, therefore, different AUs [30]. However, the presentation of the AUs and the frequency of the movements can differ or even be absent according to the species.

### 4.1. ChimpFACS

ChimpFACS was developed by Parr et al. [20] and is a system that describes 15 facial movements according to the anatomy, position, and movement of mimetic muscles. In chimpanzees, a total of 22–23 mimetic muscles have been found (e.g., *risorius*, *depressor septi*, *corrugator supercilli*, *depressor supercilli*, *sphincter colli*, and *caninus muscle*, among others, schematized in Figure 2) [4]. Although chimpanzees have similar AUs and mimetic muscles to humans (e.g., the *risorius muscle*, a structure that has only been reported in humans) [78], their anatomy is different by having more prognathic faces, elongated mouths, lower foreheads, and a flatter nasal area [21]. Moreover, Burrows et al. [79] mention that chimpanzees have thicker mimetic muscles around the oral cavity.

The 15 AUs described in chimpanzees are summarized in Table 1, considering their name, description, and musculature and comparing them with the original FACS developed for humans. An AU that is unique to the ChimpFACS is the lower lip relax (AU160) [20,21].

Further studies in chimpanzees have classified the AUs into eight categories, including bared-teeth display, pant-hoot, play face, scream, pout, and whimper [20,81,82] (Table 2) [20,82]. When evaluating the context, some of these AUs share the function and the cause. For example, bared teeth (BT) display is frequently compared with human smiles due to the morphological and functional similarities. All variants of BT display involve the movements of the *zygomaticus major muscle* [83]. Nonetheless, Kim et al. [28] found in captive chimpanzees that the meaning depends on several characteristics. One is the social ranking, where all chimpanzees exhibit BT display except the alpha male. The silent BT display, characterized by a slightly open or closed mouth with the corners retracted laterally, fully exposed teeth, and withdrawn lips, is mainly observed during affiliative subordinate-to-dominant interactions (0.98 probability). In contrast, the vocalized BT display where the mouth of the chimpanzees is partially open with retracted corners and fully exposed teeth, accompanied by high-pitched screams was present in both aggressive (probability of 1.0) and affiliative interactions (0.95). In contrast, other authors mention that BT displays are present during socio-sexual interactions between great apes as an appeasement signal to regulate tension [1].

### 4.2. OrangFACS

OrangFACS was the second AnimalFACS developed for great apes. Similar to ChimpFACS, OrangFACS comprises 17 AUs according to the musculature (Table 1) [27]. Some studies have detailed certain facial behaviors in orangutans, such as the play face (PF) associated with a relaxed open mouth (ROM). Similar to chimpanzees (AU12 + 25 + 26 or AU12 + 25 + 27) [20], in orangutans, this facial expression is described as AU10 + 12 + 25 + 27 [52]. However, differences are also present. While the authors found that no AUs are exclusively from orangutans, AU4 (an AU thought to be exclusive to humans) and AU18 (not found in chimpanzees) were clearly observed in this species [27].

As reported in chimpanzees, certain facial movements such as BT display (AU10 + 12 + 25) [27] have been associated with distinctive contexts such as fear followed by aggression in Sumatran orangutans (*P. abelii*) [84].

### 4.3. What About Gorillas?

In the case of gorillas, information regarding their facial anatomy is limited and, to date, there is not a designed FACS for the species. However, detailed facial dissections have been recently performed by Rotenstreich and Marom [4] to describe the gross and microanatomy of the supraorbital, lateral orbital, zygomatic, upper labial, and lower labial areas. By dissecting a female gorilla, 18 muscles were found, which closely resemble those reported in chimpanzees, orangutans (*P. pygmaeus*), gibbons (*Hylobates species*), baboons (*Papio*), macaques (*M. mulatta*), and humans. Significant differences were observed in gorillas, particularly in the lower facial anatomy. For example, *the levator labii superioris alaeque nasi*, the *levator labii superioris*, and the *zygomaticus minor* muscles are close together, in contrast to humans and chimpanzees. In contrast to orangutans, macaques, gibbons, and baboons, gorillas do not have a modiolar region—the area of the labial commissure. Gorillas also lacked the *risorius* muscle, a muscle found in chimpanzees and humans. Moreover, in the lower labial region, the *depressor anguli oris* muscle does not insert into the modiolus (as seen in humans and chimpanzees), but its fibers form a continuous arc that connects the maxillary canine fossa with the SMAS (a morphology observed in orangutans) [4].

In *Gorilla gorilla*, an attempt to describe the AUs was made by Dobson [51], who determined that 15 AUs were present in 13 individuals (AU1 + 2, AU9, AU10, AU12, AU13, AU14, AU15, AU16, AU17, AU18, AU22, AU23, AU24, AU25). Attempts to classify facial expressions have also been published, where a PF and full PF (FPF) are described as AU16 + 25 + 26 and AU AU10 + 16 + 25 + 26, respectively [52]. However, since there is no other study that has assessed the same AU, no comparison or confirmation is available.

The ability of each AnimalFACS to classify great apes’ facial expressions in AUs according to their underlying musculature can be used to further identify the facial changes involving exposure to emotional stimuli [20]. The current AnimalFACS is different from a facial ethogram; therefore, a single AU cannot and must not be used to represent an emotion or a mental state. The FACS helps to study in detail the movements of mimetic muscles instead of analyzing facial expressions [21], as a combination of certain AUs can produce an already known facial behavior (e.g., silent bared-teeth display) that might be part of a species facial ethogram. Although most studies focus on the identification of changes in facial expression when animals are exposed to different types of stimuli, this could help to give a more objective interpretation of the facial changes, as discussed below.

## 5. Facial Expression in Great Apes as a Possible Reflection of Their Affective Mental State

In humans, basic emotions such as anger, fear, happiness, sadness, surprise, and disgust have been identified, and some authors have suggested that emotional facial expressions are also present in nonhuman primates [85,86]. In contrast, in animals, no consensus has been reached so far on the role of facial displays in expressing emotional states as animals cannot self-report the intensity and valence of their own emotional experience [87]. However, current research in facial expression has suggested that facial movements and their expression could be a temporary and measurable indicator of the affective state (positive or negative) of an event [88].

Detailed descriptions of facial expression repertoires in great apes have been studied when animals respond to certain stimuli that are known to be positive or negative to them. Parr et al. [89] described a facial ethogram for chimpanzees that included expressions such as PF, pant-hoot, ambiguous faces, neutral expressions, scream, alert faces, pout, and whimper [20]. Although no FACS is available for gorillas, Tanner [90] mentions that this species is highly expressive, showing PF, pouts, tongue movements, and different degrees of BT displays. In further studies, the same author reported that after a gorilla expressed a PF, play bouts began within 4 s, showing the communication role that facial expression has for nonhuman primates [91].

In general, some authors state that great apes use tactile gestures more frequently than facial expressions (47.9 vs. 4.6%) [92], while others mention that vocalization is the main emotional expression method in primates [93]. The frequency of presentation and the gestures used by primates vary according to the context, such as dominance/submission, sexual, playful, parental, or affiliative behavior [92]. In the case of orangutans, they frequently use facial expressions during parental (11.9%) and agonistic interactions (11.1%). The low percentage of facial expressions in the species is also related to their social structure and environment, where orangutans are arboreal animals, making vocal cues more important to send information about social intentions and emotional states [79].

The most observed facial expression in great apes is open mouth face (OMF) [92]. It is described as the mouth completely open with full exposure of the canine teeth and the palate, without retracting the corners of the mouth [92]. Although there are variants of this facial expression and each has a different meaning, the OMF display is often related to playful contexts. Davila-Ross et al. [94] reported in orangutans that facial mimicry of OMFs within 1 s suggests a positive emotional state when observed during dyadic play bouts. Moreover, this facial expression tends to be shared between individuals who have a stronger bond or familiarity. In the case of bonobos (*P. paniscus*), Demuru et al. [95] found that animals involved in rough and gentle play exhibited OMFs to communicate a positive context, often exhibited by younger animals.

Play faces (PFs) are the most studied facial expressions related to the OMF. Great apes use two types of playful facial expression: the PF or relaxed open mouth (ROM) where only the lower teeth are exposed, and the full play face (FPF), which exposes both the upper and lower teeth, also known as OPM and considered an intense version of PF [87,96,97]. PFs are characterized by ROM, a display that is a unique trademark of play behavior that has been observed in many species of nonhuman primates [29]. All variants share the involvement of the *zygomaticus major* muscle [28] and the contraction of the *levator labii superioris muscle*, which pulls the corners of the lips backwards and upwards, opening the lips (AU12 + 25 + 26) [98].

PFs are the only primate facial behavior that shows morphological similarities with human laughing faces [29], where their lips are separated and pulled backward and upwards while dropping their jaws; they can raise their upper lip and the cheeks [99]. Indeed, in orangutans, an ROM display is associated with play context, and Waller et al. [98] have shown that when the receiver is attentive to the facial expression of the sender, the facial movements are more intense. This was observed in videotaped orangutans during spontaneous play interactions such as wrestling, hitting, or grappling. The animals maintained their OMF between 0.08 s and 10.56 s, and this time was significantly longer when the interaction was face-to-face (1.64 s ± 1.19) than when the sender did not receive direct attention (1.09 s ± 0.71). As such, the facial orientation of the receiver also plays an important role in the intensity of the AU, particularly AU27. A similar observation was made by Bresciani et al. [96] in captive groups of lowland gorillas, in whom the number of PFs/FPFs increased when both animals were looking at each other (5.54 ± 0.72). Additionally, the number of PFs/FPFs performed in each play bout was 4.236 ± 0.150, and, contrary to what was observed in other species, gorillas tend to perform ROM faces with conspecifics with whom they do not have a close bond.

In this sense, Waller and Cherry [52] reported that Western lowland gorillas use the ROM display during playful interactions. This response can also be elicited by caretakers, as shown in a study in *P. pygmaeus* that registered the facial and vocal responses of 21 orangutans to tickle-induced play by familiar caregivers. Play faces were observed in 70% of individuals, and the FPF was the most frequently observed (in 76.6% of the total play faces), followed by the bite face (15.8%), particularly in juvenile males, who tended to show more PFs than females (58 vs. 42%). Interestingly, 75% of orangutans produced silent play faces considering both sexes [97].

Palagi et al. [87] evaluated the facial expression and mimicry of captive chimpanzees and western lowland gorillas (*Gorilla gorilla gorilla*) during play fighting, finding significant differences between species that might be associated with their social dynamics. For example, gorillas performed more FPFs (0.021 ± 0.003 FPFs per s) than chimpanzees (0.013 ± 0.003 FPFs per s). Moreover, rapid facial mimicry (known as an involuntary, automatic, and rapid imitation of conspecific facial expressions [52]) prolonged play sessions in both species. This is relevant because play fighting can escalate into aggression, making it essential for primates to communicate their intentions. The difference in facial expressions between both species is also related to their social structure, where chimpanzees live in groups with several males and females forming high social bonds [100]. Contrarily, gorillas have low affiliative and inter-individual cohesion (with only one adult male and several adult females and their offspring) [101], where correct communication and facial expression are essential to avoid attacks.

On this matter, Cordoni et al. [102] reported that among playful sessions, the facial expressions of PF and FPF were present in 17.5% and were performed by the silverback male, while the rest were performed by an adult female (82.5%). Interestingly, FPF was immediately followed by play bite (76.7% of occurrence). This is also related to FPF being more common (80%) and longer during rough play as a possible way to communicate to the playmate that this is only a game and will not escalate to actual aggression [52]. This is a clear example of the importance of differentiating facial expressions because the FPF exposes both the upper and lower teeth but with a relaxed mouth, an expression that could be confused with or a mixture of the BT display known to be a signal of appeasement or submission [54,87].

The BT display is a facial expression where the lower and upper teeth are completely exposed (AU26 + 25 + 10 + 12 + 16) [20]. It could be confused with FPF; however, the corners of the mouth are retracted laterally, and the teeth are fully exposed. Additionally, the eyes are open or squinted [28]. BT displays are frequently observed during grooming, sexual interactions, or to avoid aggression and are performed by subordinate individuals when approaching a dominant individual [82]. The silent bared-teeth display (SBT) (also called grin, grimace, or fear face) is part of the facial expression repertoire of great apes [92,103] that has been observed during agonistic and submissive interactions that elicit fear and discomfort in subordinate animals [92,103,104].

A study in chimpanzees evaluated 337 BT during dyadic interactions. When considering an aggressive context, BT display was frequently directed towards dominants and during high-tension interactions (feeding) (100%), while SBT was most pronounced in a sexual context [28]. Similarly, a study in bonobos (*Pan paniscus*) showed that SBT displays occur during a sexual context (up to 22.8%) followed by affiliative interactions (up to 18.8%) in both adult and immature animals. They also found that SBT was not the most frequent facial expression during nonfeeding as a tension condition (1.3%). Similarly, vocalized BT was often present during a sexual context (48.6%) and did not increase during tension conditions (2.1%) [1].

BT screams or a screaming face with BT in great apes (AU10 + 12 + 16 + 25 + 27) [20] is a facial behavior related to fear [80,103]. Screams are exhibited during social contexts when there is nervousness, fear, and distress or during aggression bouts [105]. In Sumatran orangutans, the exposure to an audio recording of a long call produced facial expressions associated with fear, submission, aggression, and worry as part of their behavioral response to defend their territory [106]. Other studies have shown that Sumatran orangutans direct more attention to fear faces over angry and neutral faces, as well as the SBT expression during agonistic interactions [105].

Another facial expression reported in great apes is the pout face when the lips form a trumpet-like shape (AU17 + 22 + 25) [105]. This expression is mostly seen during affiliative (play behavior) and parental contexts [92], particularly in juvenile individuals [92]. Alert faces (AU16 + 25, AU25 + 26, or AU25 + 160) are frequently observed when chimpanzees respond to aggression or anxiety contexts [20,105], while pant-hoots, composed of AU22 + 25 + 26, express bivalent situations of excitement, whether a response to noises, distress, during play bouts, or food availability [105].

Several facial expressions have been identified in great apes, and external elements such as the context and individual factors such as the species, the age, the sex, or the social position within the group influence the meaning that the facial behavior has on animals. Although facial expressions can be related to a distinctive event, to date, it is uncertain whether they can express a solid emotion.

## 6. The Challenges of Assessing Emotional Facial Expressions in Great Apes

Great apes are not able to verbally convey their emotional state to humans. Some authors state that there is no strong evidence to guarantee that facial behaviors express internal states or are only produced during extreme emotional experiences [30]. Still, their neural and somatic pathways generate facial behaviors that are often associated with specific contexts that represent positive or negative stimuli (e.g., playing and agonistic interactions, respectively).

Assessing emotional responses through facial expression in primates is also challenging because the facial morphology of primates, their features, face shape, coloration, or presence of hair, could emphasize or camouflage certain facial movements [21]. For example, chimpanzee and orangutan infants tend to have bright skin circles around the eyes that could give the appearance that the eyes are fully open or the eyebrows are permanently raised [27]. While these facial features may interfere with human evaluation of facial expression, they serve as an evolutive communication trait for primates [80]. Additionally, other elements such as the environment of the animals (e.g., orangutans living in trees, where branches and leaves present obstacles to facial visualizing) could make facial evaluation difficult [27].

An alternative to further research on facial expressions to determine the underlying emotional state is to perform a comprehensive evaluation where not only facial behaviors are considered but also other elements that may indicate the level of arousal. Including physiological measures (e.g., heart rate, temperature, hormonal analysis, or behavioral assessment) could provide an exhaustive idea of the emotional display. However, as great apes (and primates in general) are not domestic species, their management demands highly controlled environments that might be stressful for the animals [93].

Currently, thermal imaging, a non-invasive method to assess changes in the surface temperature of a body as a response to sympathetic-mediated modulation, has been proposed as an alternative to evaluating emotional states [107,108]. Infrared thermography has been applied to measure emotional responses in humans, and this has been implemented in nonhuman primates. In monkey and great apes (common marmosets, capuchins, rhesus macaques, Bornean gibbons, and western lowland gorillas), Chotard et al. [109] compared two emotional states: positive during the interaction with toys and tickling; and negative during food delay and teasing. Positive stimulus caused a drop in the surface temperature of the nose tip (−0.5 °C in monkeys and −2 °C in great apes) and increases in the periorbital region (+0.4 °C in monkeys), while the negative event triggered increases in the upper lip temperature (+1.1 °C in monkeys and 0.3 °C in apes). The authors highlighted the evident thermal changes in both positive/negative contexts and mentioned that the increase in the upper lip was probably the result of blood flow increase due to tachycardia and α- and β-adrenergic influence. Moreover, the response during positive stimuli might be the result of blood flow redirection to the ocular region or increased breathing.

Similarly, decreases in the nasal temperature were reported in wild chimpanzees after exposure to conspecifics’ aversive vocalizations (−0.23 ± 0.45 °C), while non-aversive vocalizations increased ear surface temperature (+0.10 ± 0.25 °C) [110]. The authors discussed that more arousing stimuli cause a bigger blood flow shift or can increase the performance of the auditory system. This was also observed in wild adult chimpanzees during social competitive interactions, resulting in lower nasal surface temperatures (32–34 °C), in contrast to cooperative events (~33.5–34.5 °C) [111]. In the same species, Barrault et al. [112] concluded that social feeding on meat is perceived as a more stressful event (due to competition), according to the decreases in nose temperature. Nonetheless, as found by Heintz et al. [113] in lowland gorillas, thermal imaging can detect emotional arousal when exposed to positive reinforcement and cognitive tasks with familiar humans (nasal temperature decreased), but additional studies are required to determine the valence of the interaction. Additionally, there are practical limitations when evaluating thermal imagining in great apes (e.g., distance), and this needs to be considered, as well as using other parameters such as behavioral assessments [114].

## 7. Conclusions

Studying facial expressions in great apes is an intriguing topic due to their resemblance to human faces. Anatomically based studies using the human FACS as a base have shown that chimpanzees and orangutans share mimetic musculature and AUs; however, differences by species are reported (e.g., AUs exclusive to chimpanzees or the lack of a mimetic muscle). ChimpFACS and OrangFACS are reliable tools to code facial expressions objectively in different contexts. Although the FACS is different from a facial ethogram and does not infer animal emotions, making behavioral assessments when exposed to positive/negative contexts to further codify facial expressions according to AUs is a practical approach to further appraise facial behaviors in great apes as a contribution to facial emotion research.

## Figures and Tables

**Table 1:** Classification of AUs according to their name, description, and musculature in chimpanzees and orangutans.

Action Unit	Name	Description	Musculature	Chimpanzee	Orangutan
AU1	Inner brow raiser	Pulls the medial and lateral parts of the brow upwards	*Frontalis* (medial)	✘	✘
AU2	Outer brow raise	Pulls the medial and lateral parts of the brow upwards	*Frontalis* (lateral)	✘	✘
AU1 + 2	Brow raiser		*Frontalis*	✔	✔
AU4	Brow lowerer	Lowers the brow region and pulls downward the anterior part of the scalp	*Procerus* *Depressor supercilli* *Corrugator*	✘	✔
AU5	Upper lid raiser	Elevates the upper eyelid	*Orbicularis oculi*	✘	✘
AU6	Cheek raiser	Pulls the outer and upper areas of the cheeks	*Orbicularis oculi, pars orbitalis*	✔	✔
AU7	Lid tightener	Pushes the skin under the eyelids towards the nose	*Orbicularis oculi, pars palpebralis*	✘	✘
AU9	Nose wrinkle	Horizontal wrinkles above the nose	*Levator labii superioris alaeque nasi*	✔	✔
AU10	Upper lip raiser	Raises the upper lip	*Levator labii superioris*	✔	✔
AU11	Nasiolabial furrow deepener	Pulls the nasolabial furrow upwards	*Zygomatic minor*	✘	✘
AU12	Lip corner puller	Pulls the corners of the lips backward	*Zygomatic major*	✔	✔
AU13	Sharp lip puller	Pulls the corners of the lips upward without pulling them to the back	*Levator anguli oris*	✘	✘
AU14	Dimpler	Tightens the corner of the lips with a visible oblique wrinkle in the corner	*Buccinator*	✘	✘
AU15	Lip corner depressor	Pull the corners of the lips downward	*Traingularis*	✘	✘
AU16	Lower lip depressor	Pulls the lower lip down, the lips part	*Depressor labii*	✔	✔
AU17	Chin raiser	Protrudes the lips	*Mentalis*	✔	✔
AU18	Lip pucker	Pulls the lip corners medially causing the mouth opening to shrink	*Incisivii labii*	✘	✔
AU20	Lip stretcher		*Risorius*	✘	✘
AU22	Lip funneler	Lips parted and everted in outward direction	*Orbicularis oris*	✔	✔
AU23	Lip tightener	Tightens the lips causing vertical wrinkles below and above the mouth	*Orbicularis oris*	✘	✘
AU24	Lip presser	Presses the lips together, bulging them above and below	*Orbicularis oris*	✔	✔
AU25	Lips parted			✔	✔
AU26	Jaw drop		Nonmimetic muscle	✔	✔
AU27	Mouth stretch		Nonmimetic muscle	✔	✔
AU28	Lip suck	Pulls the lips inward, stretching the skin over the teeth	*Orbicularis oris*	✔	✔
AU39	Nostril compressor	Compresses the nostrils	*Depressor septi nasi, nasalis*	✘	✘
AU43	Eye closure	Closes the eyes	*Orbicularis occuli*	✔	✔
AU45	Blink	Closes and opens the eyes	*Orbicularis occuli*	✔	✔

**Table 2:** Facial expression of chimpanzees and their assigned AUs.

Facial Expression	AU
Bulging-lip display	AU17 + 24
Bared-teeth display	AU10 + 12 + 16 + 25
Scream	AU10 + 12 + 16 + 25 + 27
Pant-hoot	AU22 + 25 + 26
Relaxed open mouth	AU12 + 25 + 26
Play face	AU12 + 25 + 26
Whimper	AU12 + 22 + 25
Pout	AU22 + 25

## Data Availability

Data sharing is not applicable.
